# Economic analysis of complementary, alternative, and integrative medicine: considerations raised by an expert panel

**DOI:** 10.1186/1472-6882-13-191

**Published:** 2013-07-25

**Authors:** Ian D Coulter, Patricia M Herman, Shanthi Nataraj

**Affiliations:** 1 Health Unit, RAND Corporation, Santa Monica, CA, USA; 2 School of Dentistry, UCLA, Los Angeles, CA, USA; 3 RAND/Samueli Chair for Integrative Medicine, Santa Monica, CA, USA; 4 Samueli Institute, Alexandria, VA, USA

**Keywords:** Complementary and alternative medicine, Integrative medicine, Economic evaluation

## Abstract

**Background:**

An international panel of experts was convened to examine the challenges faced in conducting economic analyses of Complementary, Alternative and Integrative Medicine (CAIM).

**Methods:**

A one and a half-day panel of experts was convened in early 2011 to discuss what was needed to bring about robust economic analysis of CAIM. The goals of the expert panel were to review the current state of the science of economic evaluations in health, and to discuss the issues involved in applying these methods to CAIM, recognizing its unique characteristics. The panel proceedings were audiotaped and a thematic analysis was conducted independently by two researchers. The results were then discussed and differences resolved. This manuscript summarizes the discussions held by the panel members on each theme.

**Results:**

The panel identified seven major themes regarding economic evaluation that are particularly salient to determining the economics of CAIM: standardization (in order to compare CAIM with conventional therapies, the same basic economic evaluation methods and framework must be used); identifying the question being asked, the audience targeted for the results and whose perspective is being used (e.g., the patient perspective is especially relevant to CAIM because of the high level of self-referral and out-of-pocket payment); the analytic methods to be used (e.g., the importance of treatment description and fidelity); the outcomes to be measured (e.g., it is important to consider a broad range of outcomes, particularly for CAIM therapies, which often treat the whole person rather than a specific symptom or disease); costs (e.g., again because of treating the whole person, the impact of CAIM on overall healthcare costs, rather than only disease-specific costs, should be measured); implementation (e.g., highlighting studies where CAIM allows cost savings may help offset its image as an “add on” cost); and generalizability (e.g., proper reporting can enable study results to be useful beyond the study sample).

**Conclusions:**

The business case for CAIM depends on economic analysis and standard methods for conducting such economic evaluations exist. The challenge for CAIM lies in appropriately applying these methods. The deliberations of this panel provide a list of factors to be considered in meeting that challenge.

## Background

Why should we care about the economics of complementary, alternative and integrative medicine (CAIM)? Healthcare is expensive and becoming more so every year. Policy and decision makers increasingly need information on costs, as well as effectiveness and safety, in order to formulate healthcare strategies that are both clinically effective and financially responsible. In addition, a report prepared for the 2009 Institute of Medicine (IOM) *Summit on Integrative Medicine and the Health of the Public* notes that “[b]oth clinical effectiveness and cost effectiveness are required to formulate evidence-based policy [1] (p. 3)”. If CAIM is to be considered in health care policy and reimbursement decisions, its economic impact must be determined.

## Methods

On January 26 and 27, 2011, RAND Corporation, in collaboration with the Samueli Institute, convened a panel of experts to discuss what was needed to bring about robust economic analysis of CAIM. The goals of the expert panel were to review the current state of the science of economic evaluations in health, and to discuss the issues involved in applying these methods to CAIM, recognizing its unique characteristics. The panel was chosen in consultation with the Samueli Institute and was comprised of eleven scholars with experience in applied health economics in general, the economic analysis of CAIM in particular, and/or CAIM research.

Prior to convening the panel, a background paper was developed that summarized the state of the science with respect to economic evaluation of CAIM, and a list of example published studies compiled using a simple search strategy of “complementary therapies” AND “cost benefit analysis”. Studies that were full economic evaluations (i.e., those that presented costs and benefits for two or more options), or whose study designs illustrated some aspect that might be particularly relevant to CAIM (e.g., incorporating patient preferences into the allocation process) were included in the list. This background paper and list of example studies were circulated among panel members, and their comments and feedback were incorporated prior to the meeting. These revised documents (available upon request from the corresponding author) were then redistributed to panel members for their use at the meeting.

The one and a half-day panel meeting was held at RAND Corporation offices in Santa Monica, California. The morning of the first day of the panel involved general discussion about economic evaluation and its application to CAIM. Discussions during the afternoon of the first day and morning of the second day were framed around a ten-point checklist for assessing economic evaluations in health care from Drummond et al [2]. Based on panel member input, we developed a matrix of issues to be considered for each item on this checklist with emphasis on issues that are unique or particularly salient to CAIM. This matrix was used as a guide for panel discussions on the morning of the second day. We used an audiotape to record the panel proceedings during both days.

Following the panel, the authors conducted a thematic analysis, in which two researchers independently listened to the audiotape, listed the topics that were discussed, and grouped the topics into themes. The themes were then discussed and agreed upon, and panel members’ discussions on each summarized. A report was written on the results of the panel and circulated to the panel members for comment and revisions. This article presents a summary of the themes discussed and recommendations made to CAIM researchers interested in economic evaluation.

## Results

### Background on economic evaluations

Since the panellists were generally not experts in economic evaluation, the background paper and a fair amount of the panel discussion was spent summarizing and reviewing its main points. These points are briefly reviewed here.

Drummond et al. [2] define an economic evaluation as “the comparative analysis of alternative courses of action in terms of both their costs and consequences”. (p. 9) Consequences can include economic, clinical, and humanistic outcomes [3]. The specific costs and economic outcomes that should be included depend on the perspective from which the evaluation is conducted, and may include direct medical costs (such as practitioner fees or drug costs), direct non-medical costs (such as travel costs associated with receiving the therapy), and indirect costs (such as lost worker productivity). Clinical and humanistic outcomes can collectively be called health outcomes and are measured in effectiveness trials. The form in which health outcomes are reported determine the type of economic evaluation performed.

There are three main types of “full” economic evaluations. Full economic evaluations compare the costs and consequences of two or more alternatives. Partial evaluations only compare costs, or report costs and consequences for only one option.

#### Cost-effectiveness analysis (CEA)

This type of study measures the costs and economic consequences of a therapy in monetary units, and clinical outcomes in “natural” units, such as the amount by which cholesterol is lowered, or the number of life-years saved.

#### Cost-utility analysis (CUA)

Like a CEA, a CUA measures costs and economic consequences in terms of monetary units. However, health outcomes are reported in terms of a measure of overall health such as quality-adjusted life-years (QALYs). QALYs account for both morbidity and mortality by weighting life-years by the health-related quality of life in those years [4].

#### Cost-benefit analysis (CBA)

A CBA measures costs, economic consequences and health outcomes all in monetary terms. Monetary values for health outcomes can be estimated by using a variety of methods, including those that elicit willingness-to-pay (WTP) for the outcome.

The main advantage of conducting a CEA, rather than a CUA or CBA, is that these studies can use the clinical outcome measured during the effectiveness trial. However, CEA is less helpful if the treatments being compared do not affect the same health outcomes. CEA is most useful when comparing treatments that have the same one-dimensional goal—e.g., a reduction in glycosylated hemoglobin (HbA1c) [2]. CUA and CBA analyses address this issue by measuring health outcomes in terms of more general units (e.g., QALYs in case of CUA and monetary terms in the case of CBA), which allow comparisons to be made across therapies that affect different aspects of health.

### Panel themes and recommendations for each

In this section, we present a summary of the main issues discussed during the panel. The seven major discussion themes (listed in Table 1 and discussed below) are the result of thematic analysis of the panel proceedings. To increase the usefulness of these results to researchers interested in CAIM economic evaluation, references are given for some points made by the panel, and the authors have included additional information from the literature (noted as such) to further expand on others.

1. Standardization

**Table 1:** Summary of discussion themes and recommendations made by the panel

**Themes**	**Recommendations**
1. Standardization	●In order to compare CAIM with conventional therapies, the same basic economic evaluation framework and methods must be used.
2. The question, audience, and perspective	●Given the high level of self-referral and out-of-pocket payment the patients’ perspective as to important health outcomes and relevant costs should be incorporated into CAIM economic evaluations either as a separate analysis or as part of the societal perspective when that perspective is used.
3. Analytic methods	●Both the treatment and usual care arms of CAIM trials should be described in detail given the heterogeneity of treatment across practitioners.
	●To capture the preventive nature of CAIM, longer follow ups and/or modeling should be used to capture impacts beyond the treatment phase.
4. Outcomes	●Given the tendency for CAIM to treat the whole person, rather than only the targeted condition, measure and report a wide range of outcomes, and/or to use a summary measure of overall health that could capture the full range such as quality-adjusted life-years (QALYs).
	●If multiple outcomes are used, consider reporting these as a cost-consequence analysis.
5. Costs	●Given that CAIM is not often covered by health insurance (whose claims databases are the source of much of the cost data available to studies of conventional medicine) and since the relative quality of other sources of cost data (e.g., practitioner records or patient self-report) is not always clear, both sets of costs could be reported.
	●Since CAIM therapies tend to treat the whole person they may have cost impacts beyond the targeted disease. Therefore, it is recommended that overall healthcare costs be reported, either in addition to or instead of disease-specific costs.
6. Implementation	●Highlighting studies where (and the circumstances under which) CAIM can both improve health outcomes and *reduce* costs (rather than increase costs, even if still considered cost effective) might gain it more attention from policy makers.
7. Generalizability	●The first recommendation made under theme 3 above is reiterated here; this information is also needed to enable study results to be adapted to other settings.
	●As compared to conventional therapies, CAIM therapies might require more sensitivity analyses to test the robustness of results given the large variability in treatment methods, dosages, and pricing.
	●Given that patient beliefs can have a large impact on outcomes, especially in CAIM, these beliefs should be measured and reported to allow comparisons to other populations.

During the panel, there was a substantial amount of discussion about whether the methods used to perform economic evaluations of conventional healthcare options were appropriate for CAIM. In the end, the panellists generally agreed that in order to compare CAIM with conventional therapies, the same basic economic evaluation framework and methods must be used. They noted that currently there is a tendency for medical practitioners and policymakers to react to CAIM based on philosophy rather than on evidence. In order to integrate CAIM and conventional medicine, CAIM therapies will need to demonstrate success in some comparable way. Moreover, the panel noted that many of the issues discussed here also apply to evaluations of conventional medicine.

2. The Question, Audience, and Perspective

The panellists agreed that, similar to what is done in economic evaluations of conventional medicine, before beginning a study, it is crucial to determine the question that the study is intended to answer and whom that answer is intended to inform (i.e., the audience). The question will determine which alternatives to compare and the audience will determine the perspective of the evaluation. As discussed above, the perspective of the analysis determines which costs will be included. For example, if the purpose of an economic evaluation is to provide information to a health plan on the benefits of providing coverage for acupuncture for headache pain, the comparison should be between acupuncture and the therapies the plan now covers for headache, and the perspective should be that of the health plan—also known as the third-party payer perspective. Since the payer perspective is used, direct medical expenses that would be reimbursed by the payer would be included, but out-of-pocket expenses (including co-pays or deductibles) and travel costs incurred by patients would not. Panel members also noted that whenever possible, an economic evaluation should document costs to allow for analyses appropriate for more than one audience (i.e., from more than one perspective).

One issue noted by the panel as specific to CAIM, but also increasingly relevant for all of healthcare, is economic evaluation from the *patient* perspective. In practice, this means that the costs to the patient (e.g., out-of-pocket costs and travel and time costs involved with receiving the treatment) and the benefits of importance to patients are included in a separate analysis showing the patient perspective, and/or that all patient costs are included in the societal perspective. The patient perspective is especially relevant to CAIM because so much of CAIM is accessed directly through patient self-referral and paid out-of-pocket. Therefore, the patient’s perception of whether the benefits of care exceed its cost is important.

Additional information: It should be noted that the patient perspective is assumed to be included in economic evaluations from the societal perspective. The patient perspective is, for the most part, ignored in economic evaluations of conventional medicine. This lack of focus on the patient perspective could explain the inconsistencies seen in conventional evaluations using the societal perspective [5-7].

3. Analytic Methods

As discussed above, the panel generally agreed that the same methods should be used to evaluate conventional therapies and CAIM, though the methods may need to be tailored to better fit the latter. Along these lines, the panel discussed the challenges inherent in determining the effectiveness of CAIM since evidence of effectiveness is a required input for economic evaluation.

Panel members noted that historically, a significant amount of CAIM research funding has been provided for trials that illuminate the mechanism through which a therapy affects health outcomes—i.e., efficacy trials. More recently, however, research has begun to focus first on whether a therapy is effective, and then only consider the mechanisms through which it might work once it has been proven effective; however, this remains a controversial area.

Related to the real world focus of an effectiveness trial (and economic evaluation), the comparator also must represent a real world option such as “usual care”; placebo is rarely an appropriate comparator. However, panel members acknowledged that defining “usual care” could present a challenge for certain CAIM therapies such as chiropractic. In some cases chiropractic is already considered to be part of “usual care,” so if the treatment is to provide chiropractic services, it would be unclear how to define “usual care” in the control arm.

The panel agreed that the definition of the treatment arm also offers special challenges in CAIM. Care processes may differ drastically across practitioners, even more so than in conventional medicine. Therefore, it might be important to explore practitioner and treatment heterogeneity, and to document during the trial the characteristics of the treatment actually provided (i.e., treatment fidelity [8]). This documentation is important both to better communicate to others what happened during the study, and to increase the ability to replicate the results. Several panel members advocated the use of qualitative methods, including observational studies and ethnographic observation, to provide information about the care process [9].

Finally, panel members generally agreed that given the prevention focus of much of CAIM, it would be desirable to use a relatively long timeframe to measure outcomes.

Additional information: There has been much written on the challenges of measuring the health impacts of CAIM [10-13]. Because full economic evaluations require estimates of both costs and health impacts, these challenges will also apply to economic evaluations of CAIM. Note that an economic evaluation requires a measure of *effectiveness*; not *efficacy*. Efficacy “denotes how well the intended objectives are realized in ideal settings”, while effectiveness can be defined as “the extent to which [health improvements are achieved] in real practice settings [14]”.

Comparative Effectiveness Research (CER) has placed a new focus on effectiveness, and it has been given added weight by funding through NIH and in particular AHRQ (Agency for Healthcare Research and Quality) [9]. CER has variously been described as including pragmatic trials, head to head trials, and real world trials [15-19]. This trend may be especially important for CAIM therapies that are already in common use, even though the mechanisms through which they work may be poorly understood by the medical community.

Unfortunately, given the need for longer trials to capture the preventive nature of CAIM, trial participants are generally only followed for one year or less [20]. However, longer-run results can be estimated through modelling (see Herman et al. [21] for an example, and Briggs et al. for an overview of modelling methods [22]).

4. Outcomes

Because CAIM tends to address the whole person rather than target a specific symptom or disease, the panel discussed how it can have a broad range of impacts on (and beyond) health. As discussed above, CEAs (the most common form of economic evaluation) are reported as a monetary amount per some unit of health outcome, such as the cost per percentage point reduction in HbA1c for therapies targeting diabetes. One challenge identified by the panel for CAIM is that standard CEA methodology would allocate all costs to just one of what could be many different types of health (and non-health) effects seen with CAIM. For example, a CAIM therapy targeting diabetes may not only lower HbA1c, but also improve blood lipids, sleep, and overall energy. Reporting the cost-effectiveness of this CAIM therapy only in terms of a cost per point reduction in HbA1c would ignore all its other benefits and needlessly penalize this therapy in comparisons. Therefore, it is important to measure and report a wide range of outcomes, and/or to use a summary measure of overall health that could capture the full range such as quality-adjusted life-years (QALYs).

The panel also considered the possibility of developing or using an overall outcome measure that is specific to CAIM. However, two objections were raised. First, although several scales that may be more appropriate for CAIM therapies, such as the Measure Yourself Medical Outcome Profile (MYMOP) and the Arizona Integrative Outcomes Scale (AIOS), have been developed, it is not currently possible to easily translate these latter measures into QALYs—i.e., no preference weights exist for these measures. Second, if the goal is to compare CAIM to usual care, then it will be necessary to use a measure that will enable comparison with conventional therapies.

The panel discussed a number of outcomes that could be considered in economic evaluation of CAIM. Recognizing the importance of the patient perspective makes the practices and outcomes valued by the patient more critical. It should be noted that the benefits of care to the patient may extend beyond health improvement for the condition of interest, and beyond health improvement in general. For example, patients may derive value from the process of care—aka process utility [23,24]. This may come through a valued relationship with their practitioner, or through the self-empowerment achieved by actively improving their health. It was deemed unclear as to whether effects on the patient’s family should be considered. These additional patient benefits can be missed if the context and goals of CAIM and the patient perspective are not considered. The panel also noted that standard outcome measures such as QALYs may present a challenge, as this measure may mean little to patients. One panel member suggested that using methods such as conjoint analysis (i.e., a set of techniques to measure individuals’ preferences and tradeoffs across options [25]) could be helpful in measuring a broad range of outcomes.

The panel also discussed a number of other outcome measures that could be considered. These include: non-medical outcomes such as overall quality of life (rather than only health-related quality of life as is found in QALYs) or increased worker retention; risk of future events (e.g., cardiovascular events) estimates could provide a substitute for extending study duration to measure actual incidence; equity (i.e., fairness) such as whether an underserved group receives access to a therapy; and modifiable genetic markers.

Additional information: Quality-Adjusted Life-Years (QALYs) provide a summary measure of overall health by combining health-related quality of life with length of life. A CUA is an economic evaluation that compares cost to some measure of “utility” (roughly, overall satisfaction with health) such as a QALY. The panel discussed the fact that if QALYs are used to measure outcomes, then researchers should carefully choose the most appropriate instrument. The two QALY measures used most often in economic evaluations of CAIM [5,20] are the EQ-5D [26] and the SF-6D [27,28]. The SF-6D is calculated using a subset of items from either the SF-36 [27] or SF-12 [28] instruments. The EQ-5D appears to be less sensitive to changes in the upper end of the health utility range (i.e., it exhibits ceiling effects), while the SF-6D appears to be less sensitive in the lower end of the health utility range (i.e., it exhibits floor effects) [29]. Therefore, the SF-6D may be more sensitive to changes in health for populations who are generally well—the populations often targeted by CAIM.

For a discussion on how impacts on the family could be included in an economic evaluation see Bonomi et al., 2005 [30].

One form of reporting an economic evaluation with a wide range of outcomes is called a cost-consequence analysis (CCA). A CCA allows decision makers to select from a list the components of costs and health benefits most relevant to their perspective and needs [2,31,32]. Basically, instead of (or in addition to) reporting an incremental CEA or CUA ratio, the economic evaluation reports estimates for each cost component (e.g., direct outpatient visit costs, medication costs, participant travel costs, indirect/productivity costs, etc.) and for each health effect (e.g., improvement in each biomarker, stress reduction, increase in self-efficacy, changes in quality of life, absentee days reduced, QALY gains, etc.) for each arm of the study. See Dzator et al., 2004, for an example [33]. This approach may be important for CAIM for two reasons. First, it allows measurement and acknowledgment of a full range of health (and non-health) impacts seen in CAIM [34]. Second, some authors believe that this method of reporting outcomes may improve decision makers’ use of economic information [31].

5. Costs

The panel identified a number of challenges that arise when measuring costs for CAIM therapies. First, in contrast to conventional medicine where allowed charges tend to be dictated by health plans, the prices charged for CAIM therapies tend to be more heterogeneous, and typical or average prices on an aggregate (e.g., state or national) basis are generally unknown.

Second, since CAIM therapies are typically not covered by insurance plans, claims data—the source of cost data for most economic evaluations of conventional medicine—are not generally available for the CAIM therapy under study. Options to overcome the lack of claims data include patient-reported costs or practitioner-generated medical or clinic records. Panel members indicated that there are potential drawbacks to both methods. Resource use (cost) data reported by patients and practitioners often do not agree, and it is not clear which should be viewed as more accurate. This issue is compounded by the fact that prices are heterogeneous for CAIM services, so it is difficult to determine a reasonable range for cost data. In addition, a good coding of disease states is needed in order to accurately ascribe costs. CAIM disease classifications (many of which are derived from non-Western traditional practices) may not follow conventional classifications.

The panel suggested that if both patient and practitioner data are available, and it is not clear which is more accurate, both sets of costs could be reported in the study, one as the base case and the other in a sensitivity analysis. In addition, it is also possible to use expert opinion as a source of cost data.

One issue that arises in all economic evaluations is whether to include overall (all) healthcare costs, or only disease-specific costs for the targeted condition [5,35]. The panel agreed that overall costs would be more relevant to CAIM studies, since these therapies tend to treat the whole person rather than a specific symptom or disease. The panel recommended that all costs that could be measured within the study budget should be included in the study.

6. Implementation

There were several discussions regarding implementation: how study results are translated into practice. Panel members noted that it takes a long time for evidence to change practice and that this might present an especially large barrier for CAIM since it is, by definition, outside the conventional healthcare system. One discussion centered on the question of whether the cost-effectiveness of a therapy actually makes a difference in policymaking. Panellists believe that highlighting CAIM therapies that can produce cost savings, versus those that might be considered cost-effective (i.e., improve health, but also increase costs), might be particularly important to gain attention in the policy context.

Related to this topic, economic evaluation offers the opportunity to determine whether a CAIM therapy is complementary (i.e., is an “add-on” to) conventional care or alternative (i.e., replaces it). It is not always clear whether a particular therapy will be used as a replacement for, or in addition to, usual care, and the cost-effectiveness of the therapy can differ depending on how it is used. For example, a study of several CAIM therapies commissioned by the National Institute of Complementary Medicine (NICM) in Australia found that acupuncture was cost-effective for chronic low-back pain when used as a complement, but not as a substitute, for standard care [36].

7. Generalizability

During a number of the discussions regarding costs, outcomes, and other topics, the issue of generalizability was mentioned. Panellists indicated that there is a lack of standardization among CAIM therapies: for example, what constitutes an acupuncture treatment can vary widely between practitioners and even between patients of the same practitioner. The panel recommended consulting a representative body for the therapy being studied to develop an idea of what the profession considers reasonable treatment. They also emphasized the need to document the treatments that are actually performed during a study, in order to enable others to replicate the treatment.

Panel members discussed the fact that both conventional and CAIM medicine involve complex treatments even though this might not be as evident in conventional medicine. This is because most trials only look at changing one component of a complex treatment at a time. When comparing conventional and CAIM treatments, a whole systems approach may be necessary to capture complexity on both sides. Similarly, in describing the treatment and control groups, it may be necessary for the researcher to supply some background on the two different systems of care under which they are provided. The systems of care may differ not only in the treatments they use, but also in terms of the cultural, professional, political, and economic context in which the treatments are provided. For example, whether the care is provided in a for-profit versus not-for-profit environment, the professional culture of the practitioners, and the traditional context of providing treatment may also be important.

One panel member also pointed out that since a “usual care” control group should reflect current practices, which may differ across care settings, the intended audience of the study could also affect the type of care provided in the control arm. The panel agreed that regardless of which option is chosen, and for transferability, it is important that the study fully document what care was actually provided in both arms of the trial.

To address the issue of transferability (“generalizability”), the panel provided several recommendations. First, since costs and delivery methods vary greatly, the economic evaluation should at least list disaggregated costs, and should present separate values for the amount of each resource used and the cost of the resource, so that different audiences can tailor the cost estimates to their settings. Second, the Panel on Cost-Effectiveness in Health and Medicine [14,37] recommended that all economic evaluations conduct sensitivity analyses to test the robustness of the results. The panel convened by RAND and the Samueli Institute suggested that CAIM therapies might require more sensitivity analyses than conventional therapies given the large variability in treatment methods, dosages, and pricing. Third, panel members discussed the idea of heterogeneity among patients. One specific source of heterogeneity mentioned was that of beliefs. Patient beliefs may play a role in effectiveness [11,38-42]. Since the beliefs of patients enrolled in an effectiveness trial may be different from those in the population as a whole, the outcomes measured in the study may not be generalizable to the population. The panel recommended that targeting a heterogeneous population for an effectiveness trial could be helpful, since it would allow researchers to test the robustness of the effects across different types of patients, and might allow a subgroup analysis to be performed.

Finally, the importance of scalability was also discussed; panel members indicated that treatment costs could be either higher or lower if the treatment were implemented on a large scale.

Additional information: Although health outcomes are, to some extent, considered generalizable across settings, economic outcomes usually are not [43]. This may be because human physiology and psychology are more consistent and replicable across locations and settings than are resource availability, practice patterns and relative prices. Because of this, one goal in economic evaluation is to ensure the *transferability* of study results—i.e., to provide enough study detail so that results can be adapted (usually via modeling) to other settings [44]. Studies have shown that the aspect of setting which has the most effect on costs is unit price [45]. Fortunately, the problem of price variation across settings is the easiest to handle methodologically, through the separate reporting of resource use and unit costs [46,47].

In contrast to the provision of a specific drug or therapy to treat a specific symptom, much of CAIM consists of complex treatments that address the whole person. Beckman et al. [48] and Bell et al. [49] have argued that this contrast necessitates the use of a systems theory approach to study not just health outcomes, but overall wellness along multiple (bio-psycho-socio-spiritual) dimensions as well as the relationship between the patient and the practitioner. In a similar vein, Ritenbaugh et al. [50] and Verhoef et al. [51] have advocated the use of whole systems research, which “encompasses investigation of both the processes and the outcomes of complex health care interventions [50]”.

## Discussion

Economic analysis of CAIM will become increasingly important as the healthcare system struggles to contain costs. Traditionally CAIM providers have developed a direct-to-patient fee-for-service economic model that has sustained their development and growth. Much of integrative medicine has depended extensively on philanthropy and research grants to fund its growth [52]. But CAIM will increasingly face the challenge of finding a sustainable economic model; in addition, as CAIM therapies compete more with biomedicine and each other, they will need economic analysis, along with evidence about efficacy and effectiveness, to support their inclusion in health care systems and funding programs.

This study has several limitations. The first is that although RAND and Samueli Institute staff were successful in assembling a wide range of experts for the panel, the results of the panel are still limited by who did (and did not) participate. Related to this, the topics included in this paper are limited by those topics the panel raised and chose to discuss. Finally, although the background paper and list of example studies met their intent of providing a common starting place for panel members regarding their discussions, by necessity they could not provide an exhaustive education on economic evaluation to those who were CAIM researchers, nor one on CAIM for those well-versed in economics. Therefore, likely more time than originally intended in the limited panel schedule was spent on mutual education. However, it is possible that this mix is also responsible for themes and recommendations that would not otherwise have come up.

## Conclusions

Absent evidence of economic impacts, it is difficult to see how the business case can be made for CAIM. The methods for economic analysis exist. The challenge for CAIM lies in appropriately applying these methods.^a^ The deliberations of this panel provide a list of factors to be considered in meeting that challenge.

## Endnote

^a^In response to this panel and its outcomes the Samueli Institute has published a handbook describing the methods needed for the economic evaluation of CAIM. It is titled “*Evaluating the Economics of Complementary and Integrative Medicine”,* and is available from Amazon.

## Competing interests

The authors declare that they have no competing interests.

## Authors’ contributions

IDC was the principal investigator for the expert panel project and supervised and participated in the thematic analysis. PMH was a member of the expert panel; and PMH and SN participated in the writing and editing of the background documents and panel report. All authors read and approved the final manuscript.

## Pre-publication history

The pre-publication history for this paper can be accessed here:

http://www.biomedcentral.com/1472-6882/13/191/prepub
